# Comparative Pathogenesis of Alkhumra Hemorrhagic Fever and Kyasanur Forest Disease Viruses in a Mouse Model

**DOI:** 10.1371/journal.pntd.0002934

**Published:** 2014-06-12

**Authors:** Bevan Sawatsky, Alexander J. McAuley, Michael R. Holbrook, Dennis A. Bente

**Affiliations:** 1 Department of Microbiology & Immunology, University of Texas Medical Branch, Galveston, Texas, United States of America; 2 Galveston National Laboratory, University of Texas Medical Branch, Galveston, Texas, United States of America; 3 Department of Pathology, University of Texas Medical Branch, Galveston, Texas, Untied States of America; 4 National Institute of Allergy and Infectious Diseases, Integrated Research Facility, Frederick, Maryland, United States of America; Centre for Cellular and Molecular Biology (CCMB), India

## Abstract

Kyasanur Forest disease virus (KFDV) and Alkhumra hemorrhagic fever virus (AHFV) are genetically closely-related, tick-borne flaviviruses that cause severe, often fatal disease in humans. Flaviviruses in the tick-borne encephalitis (TBE) complex typically cause neurological disease in humans whereas patients infected with KFDV and AHFV predominately present with hemorrhagic fever. A small animal model for KFDV and AHFV to study the pathogenesis and evaluate countermeasures has been lacking mostly due to the need of a high biocontainment laboratory to work with the viruses. To evaluate the utility of an existing mouse model for tick-borne flavivirus pathogenesis, we performed serial sacrifice studies in BALB/c mice infected with either KFDV strain P9605 or AHFV strain Zaki-1. Strikingly, infection with KFDV was completely lethal in mice, while AHFV caused no clinical signs of disease and no animals succumbed to infection. KFDV and high levels of pro-inflammatory cytokines were detected in the brain at later time points, but no virus was found in visceral organs; conversely, AHFV Zaki-1 and elevated levels of cytokines were found in the visceral organs at earlier time points, but were not detected in the brain. While infection with either virus caused a generalized leukopenia, only AHFV Zaki-1 induced hematologic abnormalities in infected animals. Our data suggest that KFDV P9605 may have lost its ability to cause hemorrhagic disease as the result of multiple passages in suckling mouse brains. However, likely by virtue of fewer mouse passages, AHFV Zaki-1 has retained the ability to replicate in visceral organs, cause hematologic abnormalities, and induce pro-inflammatory cytokines without causing overt disease. Given these striking differences, the use of inbred mice and the virus passage history need to be carefully considered in the interpretation of animal studies using these viruses.

## Author Summary

Kyasanur Forest disease virus (KFDV) and Alkhumra hemorrhagic fever virus (AHFV) are tick-borne flaviviruses that cause severe hemorrhagic disease in humans. The pathogenesis of the disease is still not very well understood mostly due to the lack of suitable animal models. Despite sharing a high degree of genetic sequence similarity, KFDV replicates primarily in the brain and is uniformly lethal for BALB/c mice. In contrast, AHFV does not cause clinically overt signs in mice, replicates in the visceral organs, and induces pro-inflammatory cytokines and hematological changes. Given the striking differences in pathogenesis and tissue tropism, the use of inbred mice as well as the passage history of the virus needs to be carefully considered in the interpretation of animal studies using these viruses.

## Introduction

Kyasanur Forest disease virus (KFDV) and Alkhumra hemorrhagic fever virus (AHFV) are tick-borne flaviviruses that cause severe hemorrhagic disease in humans. KFDV and AHFV share a high degree of genetic sequence similarity (>90% amino acid identity for the E glycoprotein) despite occupying very different ecological niches [1]–[4]. KFDV was first isolated in 1957 [5]–[7] and causes outbreaks with 400–500 cases annually in Karnataka State, western India. AHFV was first isolated in 1995 [8]–[10] and has caused recent outbreaks of febrile disease in Saudi Arabia [11], [12], as well as illness in travellers returning to Europe from southern Egypt [13]. KFDV infection is primarily associated with bites from *Haemaphysalis* ticks [14], [15], while AHFV is thought to be transmitted by *Ornithodoros savignyi* and *Hyalomma dromedarii* ticks [16], [17]. Both viruses have case fatality rates of up to 20% [reviewed in 18], and work with infectious material is restricted to biosafety level 4 (BSL4) laboratories in North America.

Flaviviruses in the tick-borne encephalitis (TBE) complex typically cause neurological disease in humans. The TBE complex includes human pathogens such as tick-borne encephalitis virus (TBEV), Powassan virus (POWV), and Louping ill virus (LIV), as well as a number of other viruses that are apathogenic in humans [19]. TBEV, POWV, and LIV cause encephalitis of varying severity in humans, but some members of the TBE complex, such as Omsk hemorrhagic fever virus (OHFV), cause predominantly hemorrhagic disease with very little neurological involvement [20], [21]. KFDV and AHFV typically cause hemorrhagic disease as well [6], [8], [19], [21], but there is some evidence of central nervous involvement in infections by KFDV [22], [23] and AHFV [10], [11], [24]. Tick-borne flaviviruses therefore cause a spectrum of disease ranging from neurological to hemorrhagic manifestations, and KFDV and AHFV may occupy an intermediate disease phenotype between TBEV and OHFV. This question needs to be addressed in a small animal model. Furthermore, regulatory bodies such as the World Health Organization (WHO) require that flavivirus countermeasures have to be initially evaluated in a mouse model [25]. In recent laboratory studies, mice have been successfully used as a model for OHFV disease that generally follows the clinical presentation seen in humans [26]–[28]. However, no studies of KFDV pathogenesis in small animal models have been published in the past decade. Older publications from the 1960s and 70s sought to evaluate lethality of KFDV in wild-caught mammals from southwestern India and surrounding regions, but the amount of information contained in these studies is rather limited [29]–[35]. The body of scientific literature on the related AHFV is even smaller, and no animal studies have been published.

To evaluate the utility of existing mouse models for tick-borne flavivirus pathogenesis studies, we performed serial sacrifice studies of immunocompetent mice infected with either KFDV or AHFV to determine the tissue distribution of these viruses and to assess the physiological effects of infection. Although genetically closely related, KFDV and AHFV have very different clinical and virological presentations in BALB/c mice. Our data show that KFDV replicates primarily in the brains of infected animals, while AHFV replicates in visceral organs (kidneys, spleen, and liver) that are commonly associated with hemorrhagic diseases. Given the striking differences in pathogenesis and tissue tropism, the use of inbred mice as well as the passage history needs to be carefully considered in the interpretation of animal studies using these viruses.

## Methods

### Cells, viruses, and infections

Vero E6 were maintained in Dulbecco's modified Eagle's medium (DMEM) containing 5% fetal bovine serum (FBS) and BHK21 clone 13 cells were maintained in minimal essential medium (MEM) containing 10% FBS at 37°C with 5% CO_2_. All virus isolates were obtained from the World Reference Center for Emerging Viruses and Arboviruses (WRCEVA), which is housed at the University of Texas Medical Branch (UTMB), Galveston, Texas. KFDV P9605 (Genbank accession number JF416958) had 9 suckling mouse brain (SMB) passages and 2 Vero E6 passages; AHFV 200300001 (AHFV 2003; accession number JF416954) had 1 SMB passage and one Vero E6 passage; AHFV Zaki-1 (accession number JF416956) had one SMB passage, 2 mouse brain passages, 4 Vero passages, and 3 Vero E6 passages; and OHFV Guriev (accession number AB507800) had at least 27 SMB passages and one Vero E6 passage. All infections were performed under biosafety level 4 (BSL4) conditions at the Galveston National Laboratory (GNL), UTMB. Virus stocks were grown in Vero E6 cells. Titrations were performed using BHK21 cells by limiting dilution in MEM containing 5% (v/v) FBS and are expressed as the 50% tissue culture infectious dose (TCID_50_).

For growth kinetics, 5×10^5^ cells per well were seeded into 12-well dishes and were allowed to attach. The respective viruses were added at a multiplicity of infection (MOI) of 0.01 (5×10^3^ TCID_50_ per well) in a total volume of 500 µl per well. After incubation at 37°C for 1 h, virus inoculum was removed and replaced with 500 µl per well of fresh MEM containing 5% FBS. Beginning at 1 day post-infection, culture supernatants were harvested every 24 h and centrifuged for 5 min at 500×*g*. The clarified supernatants (∼500 µl) were transferred to fresh tubes and stored at −80°C. Cell-associated virus was collected by scraping the cell monolayers into 500 µl of fresh MEM containing 5% FBS. The resuspended cell fractions were stored at −80°C.

### Ethics statement

All animal procedures were reviewed and approved by UTMB's Institutional Animal Care and Use Committee (IACUC) in strict compliance with the Guide for the Care and Use of Laboratory Animals of the National Institutes of Health.

### Mouse infections and clinical evaluation

Four- to six-week old female BALB/c mice (strain code 028) were purchased from Charles River (Wilmington, Massachusetts). Subdermal transponders IPTT-300 measuring body temperature (Biomedic Data Solutions, Seaford, Delaware) were implanted. Subsequently, animals were moved into the ABSL-4 in the GNL and allowed to acclimate for 5 days before challenge. Mice were kept under barrier conditions in individually ventilated micro-isolator cages at five mice per cage (Tecniplast, Buguggiate, Italy) with corn-cob beading. Food and water was provided ad libitum and environmental enrichment material such as nestles were provided. All animal procedures were reviewed and approved by UTMB's Institutional Animal Care and Use Committee and in strict compliance with the guide for the care and use of laboratory animals. Mice were infected via the footpad with 2×10^3^ TCID_50_ per animal (20 µl total volume) or by intraperitoneal infection with 10^3^–10^4^ TCID_50_ per animal of virus diluted in serum-free MEM. Uninfected control animals were injected with serum-free MEM. Blood was collected by intracardial terminal bleeds in EDTA tubes. The whole blood was centrifuged for 5 min at 9,300×*g* and the resulting plasma was removed to fresh cryovials.

### Quantification of virus in tissues

For quantification of virus in post-mortem tissue specimens, a maximum of 0.3 g of tissue was homogenized in 0.6–1.0 ml of MEM containing 5% FBS for 2 rounds of 2.5 min each at 30 cycles s^−1^ using a Tissuelyser II homogenizer (Qiagen), followed by a 30 s spin at 9,300×*g* to pellet debris. The virus titers in tissue lysate supernatant and plasma samples were determined by limiting dilution and are expressed as TCID_50_ per gram of tissue or milliliter of plasma. Additional samples of each tissue were collected in PBS-buffered 4% paraformaldehyde. The fixed organs were kept at 4°C in the BSL4 laboratory, after which the fixative was changed completely and the tissue samples were brought out of the BSL4 according to institutional UTMB operating procedures.

### Hematology and clinical chemistry

For complete blood counts, 25 µl of EDTA blood was used. All cell counts were quantified using HemaVet 950FS hematology analyzer equipped with software to measure white blood cell count, red blood cell count, platelet count, hemoglobin concentration, hematocrit, mean corpuscular volume, mean corpuscular hemoglobin, and mean corpuscular hemoglobin concentration. For clinical chemistry, 100 µl of lithium heparin plasma was used. Analysis was performed using a VetScan2 Chemistry Analyzer (Abaxis Inc., Sunnyvale, CA, USA), which provides a complete diagnostic panel that includes albumin, alkaline phosphatase, alanine aminotransferase, amylase, total bilirubin, blood urea nitrogen, calcium, creatinine, glucose, and potassium. These hematological and clinical chemistry analyses were performed using whole blood in the BSL4 laboratory.

### Histopathology and immunohistochemistry

Brain, spleen, liver, kidney, and lung were harvested during necropsy, fixed in 10% neutral buffered formalin, and removed from the BSL4 based on Galveston National Laboratory (GNL) inactivation procedures. Tissues were then routinely processed by UTMB's Research Histopathology Core, cut, and stained with hematoxylin and eosin (H&E) for histopathologic examination. For immunohistochemistry, 5 µm sections were cut, air dried overnight, and placed into a 60°C oven for 1 h. The deparaffinized and rehydrated sections were quenched for 10 min in aqueous 3% hydrogen peroxide and rinsed in deionized water. Epitopes were retrieved using Biocare's rodent decloaking solution at 98°C for 40 min. Once slides were cooled, they were placed into Tris buffered saline containing 0.05% Tween-20 (TBS-T) for 5 min. Slides were blocked for 30 min with Rodent Block M from the MM HRP-polymer kit (Biocare Medical, USA), and were then rinsed with TBS-T. The slides were incubated with mouse ascites fluid antibodies against KFDV strain P9605 (R157 HMAF) and AHFV strain Zaki-1 (R204 HMAF), both kindly provided by Dr. T. Ksiazek (WRCEVA, UTMB) at a dilution of 1∶1000 overnight at 4°C. Slides were rinsed with TBS-T, incubated for 20 min with MM polymer-HRP, and rinsed again in TBS-T. Betazoid DAB Chromogen Kit (Biocare Medical, USA) was used as the substrate chromogen and the slides were counterstained with Gill's hematoxylin.

### Quantification of cytokines in mouse tissues

To determine cytokine levels in the plasma and tissues of mice, 25 µL of plasma or organ homogenate was run in duplicate with a Bio-Plex Pro Mouse Cytokine Assay kit (Bio-Rad) in the BSL4 laboratory based on the manufacturer's instructions. The kit simultaneously quantifies interleukin (IL)-1β, IL-5, IL-6, IL-10, IL-13, interferon (IFN)-γ, monocyte chemotactic protein (MCP)-1 (also known as CCL-2), and tumor necrosis factor (TNF)-α. The samples were run on the Bio-Plex 200 (Bio-Rad) and analyzed using the Bio-Plex Manager software version 6.0. Sample groups were compared by 1-way analysis of variance (ANOVA) with Tukey post-test using GraphPad Prism version 5.03. Levels of statistical significance are given as either *p*<0.05 or 0.01.

## Results

### Tick-borne flaviviruses have different *in vitro* growth profiles

**Figure 1:**
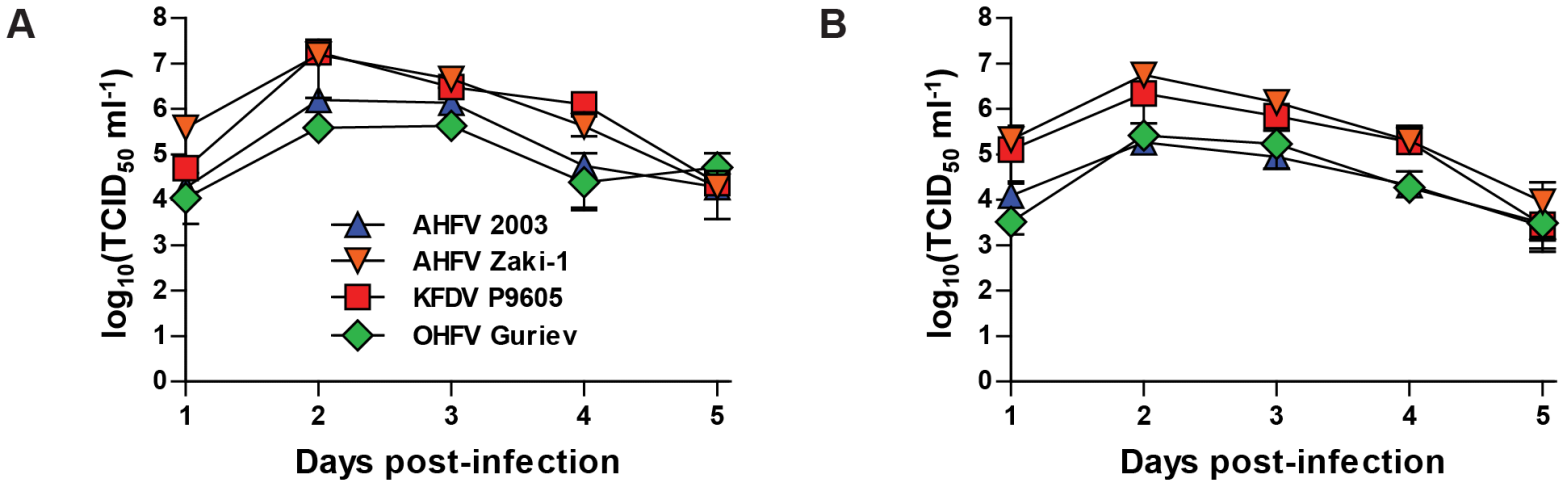
*In vitro* growth kinetics of tick-borne flaviviruses. BHK21 cells were infected with either OHFV Guriev, KFDV P9605, AHFV 2003, or AHFV Zaki-1 at an MOI of 0.01, and supernatant (A) and cell-associated (B) fractions were harvested daily for 5 days. Titers are expressed as TCID_50_ ml^−1^, and error bars represent standard deviations.

We performed growth kinetics experiments with OHFV Guriev, KFDV P9605, AHFV 200300001 (AHFV 2003), and AHFV Zaki-1 by infecting BHK21 cells at an MOI of 0.01 and followed the virus titers daily. All viruses reached peak replication in the supernatant (Figure 1A) and cell-associated (Figure 1B) fractions at day 2 post-infection, and titers decreased thereafter. There was approximately 5- to 10-fold more virus found in the supernatant than in cell-associated samples. The growth patterns of AHFV Zaki-1 and KFDV P9605 were very similar in both the supernatant (Figure 1A) and cell-associated fractions (Figure 1B), whereas AHFV 2003 was more comparable to OHFV Guriev (Figures 1A and 1B). The cytopathic effects were similar for all viruses (data not shown). Taken together, these data indicate that none of the viruses have obvious growth defects.

### AHFV does not cause clinical disease in mice

**Figure 2:**
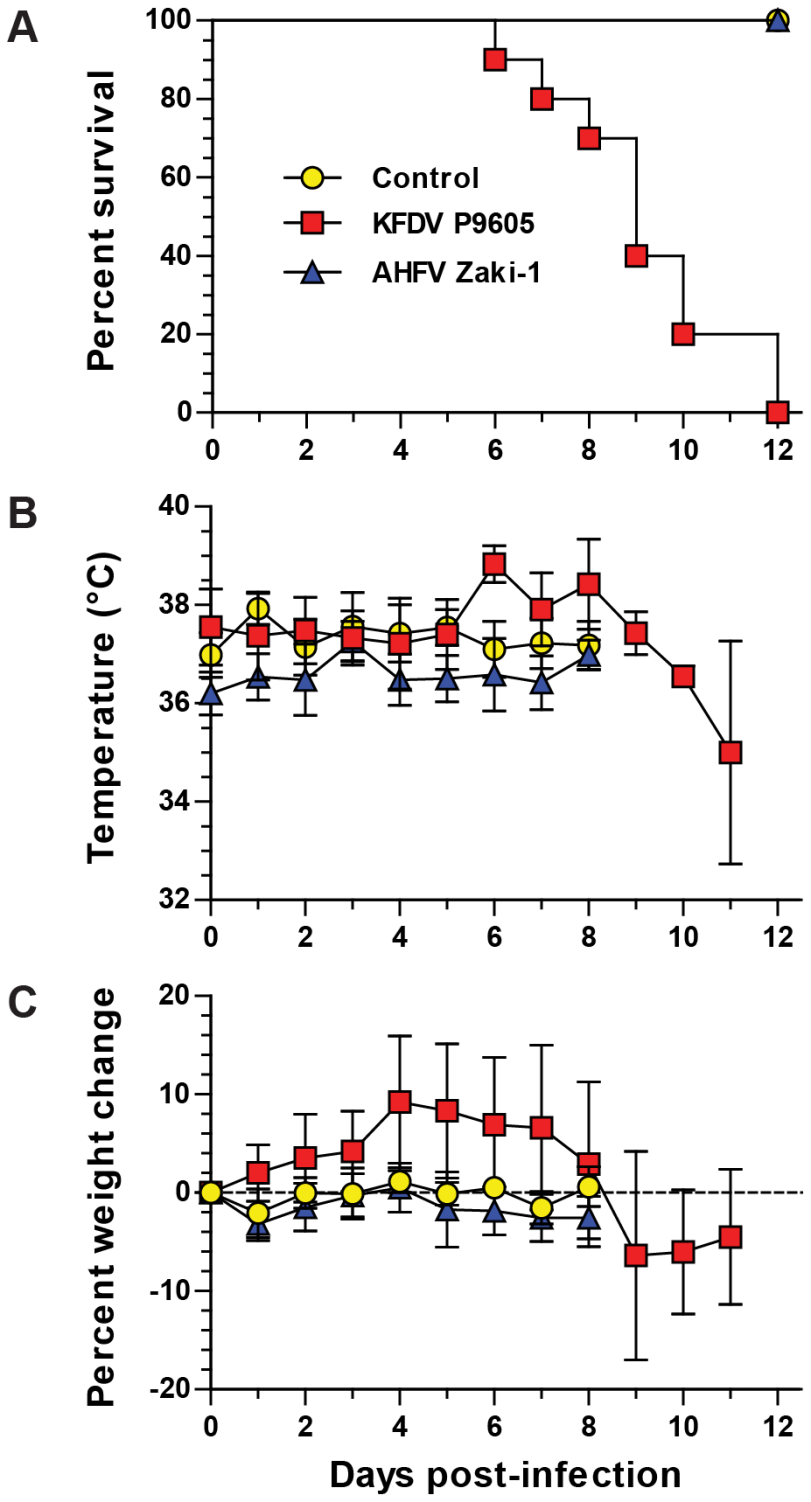
Virulence of KFDV and AHFV in mice. Groups of 5 mice were infected via footpad inoculation with 2×10^3^ TCID_50_ per animal of either KFDV P9605, AHFV 2003, or AHFV Zaki-1. (A) Survival curve. Death of an animal is indicated by a step down on the curve. (B) Temperature. Mice were implanted with subdermal transponders as detailed in the Materials and Methods, and the temperature was monitored daily. (C) Weight change. Mice were weighed daily, and the change in weight is shown as a percentage of the initial study weight on the day of infection. Error bars in (B) and (C) represent standard deviations.

We next determined the lethality of KFDV P9605, AHFV Zaki-1, and AHFV 2003 by infecting groups of 5 BALB/c mice with 10^3^ TCID_50_ per animal via footpad inoculation. BALB/c mice have been used in prior studies of tick-borne flavivirus infection and disease [26], [27]. KFDV P9605 was uniformly lethal, and animals died between days 6 and 12 post-infection (Figure 2A). Animals typically developed ruffled fur and hunched posture the day before death (data not shown) and had elevated temperatures and weight loss beginning at day 6 (Figures 2B and 2C). In contrast, mice infected with AHFV 2003 (data not shown) or AHFV Zaki-1 did not succumb to infection (Figure 2A), nor did they exhibit any signs of disease (data not shown) or significant weight loss (Figure 2C). AHFV Zaki-1 animals had slightly elevated temperatures on day 3 post-infection, but then returned to normal levels for the duration of the study (Figure 2B). We also infected mice with AHFV 2003 at doses of 10^4^ and 10^3^ TCID_50_ via the footpad and intraperitoneal route, respectively, but neither route of infection was lethal (data not shown).

### Tissue distribution of KFDV P9605 and AHFV Zaki-1 in mice

**Figure 3:**
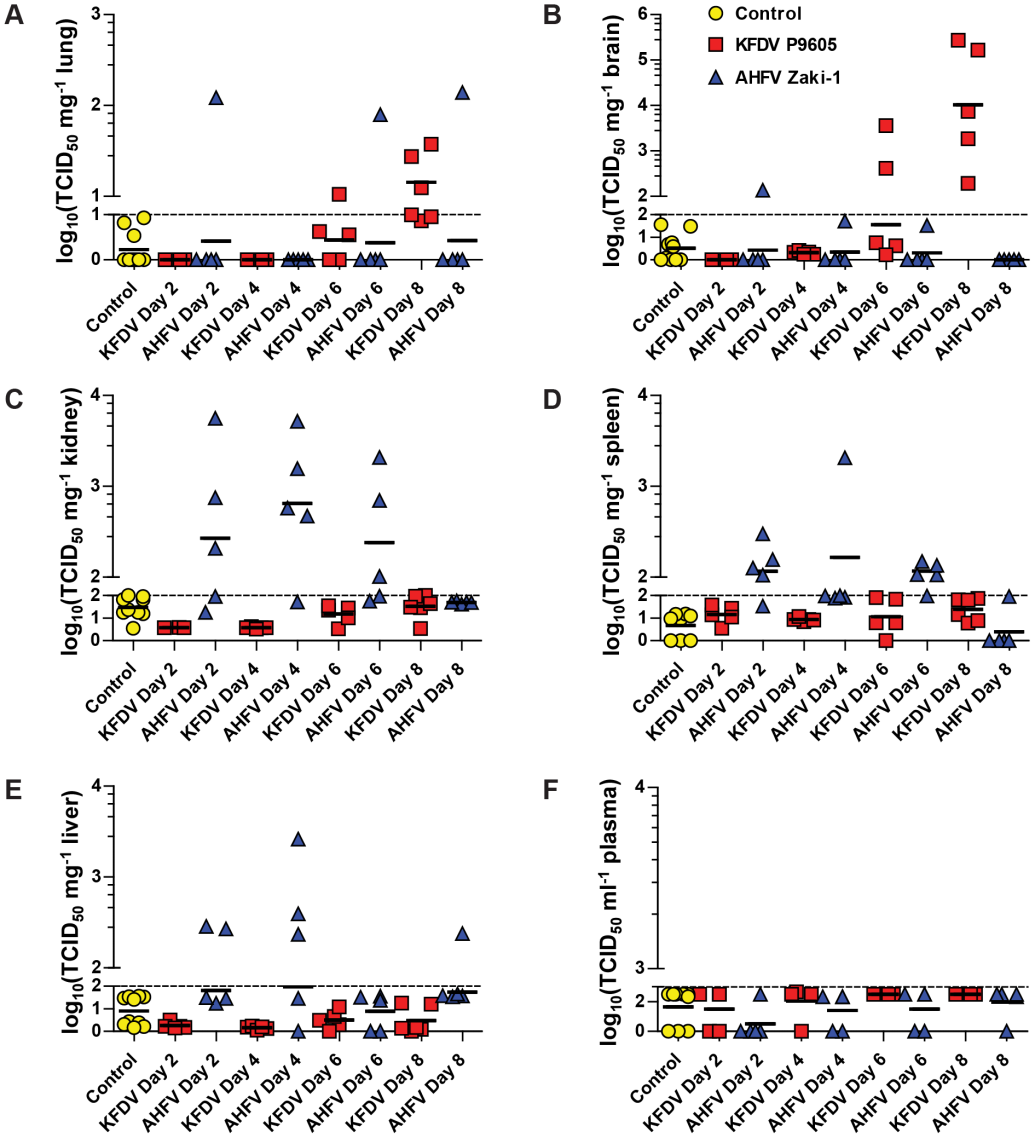
Detection of virus in organs. Groups of 20 mice were infected with 2×10^3^ TCID_50_ per animal of either KFDV P9605 or AHFV Zaki-1 via footpad inoculation. Two additional control groups of 5 mice each were inoculated with serum-free MEM. Five mice from each group were sacrificed at days 2, 4, 6, and 8 post-infection, and the controls groups were sacrificed at the end of study. Organs were harvested and homogenized as described in the Materials and Methods, and homogenates were titrated on BHK21 cells by limiting dilution. Results for the lung (A), brain (B), kidney (C), spleen (D), liver (E), and plasma (F) are shown. Each symbol represents one animal. Background cut-offs of 10^1^ TCID_50_ mg^−1^ (lung in [A]), 10^2^ TCID_50_ mg^−1^ (brain, kidney, spleen, and liver in [B–E]), and 10^3^ TCID_50_ ml^−1^ (plasma in [F]) were applied to organ and plasma titrations based on the control groups, and are shown as dashed lines on the lower axes of the respective graphs. Note in (F) that no animals were viremic. Each symbol represents one animal, and geometric means are indicated by horizontal black lines.

In order to demonstrate that animals were infected and the evaluate virus dissemination in infected mice, we performed serial sacrifice studies of BALB/c mice infected by footpad inoculation with 2×10^3^ TCID_50_ per animal of either KFDV P9605 or AHFV Zaki-1. We chose to use KFDV P9605 and AHFV Zaki-1 for detailed pathogenesis studies because they had similar growth kinetic profiles in cell culture (see Figure 1). Groups of five mice were sacrificed on days 2, 4, 6, and 8 post-infection and organ homogenates were titrated to determine the presence of infectious virus. Mice infected with KFDV P9605 had small amounts of virus in the lung on day 6, which increased on day 8 (Figure 3A). The same pattern was observed in the brain, where several animals were positive on day 6, but all animals had virus in the brain on day 8 (Figure 3B). Virus was not detected in the homogenates of the kidney, spleen, or liver from animals infected with KFDV P9605 (Figure 3C, 3D, and 3E). AHFV Zaki-1 was only detected in the lung and brain of 1 of 5 infected mice on day 2 post-infection, but others were negative throughout the study (Figures 3A and 3B). However, relatively high amounts of AHFV Zaki-1 were found in kidney, spleen, and liver (Figures 3D, 3D, and 3E). Peak titers in the kidney were detected on day 2 post-infection, but declined during the study and virus was cleared by day 8 (Figure 3C). Few animals had virus in the spleen and liver, with the highest titers detected on day 4 post-infection, and were then cleared by day 8 (Figures 3D and 3E); we did not detect virus in the plasma (Figure 3F).

### Histopathology of KFDV P9605 and immunohistochemical detection in the brain and lung

**Figure 4:**
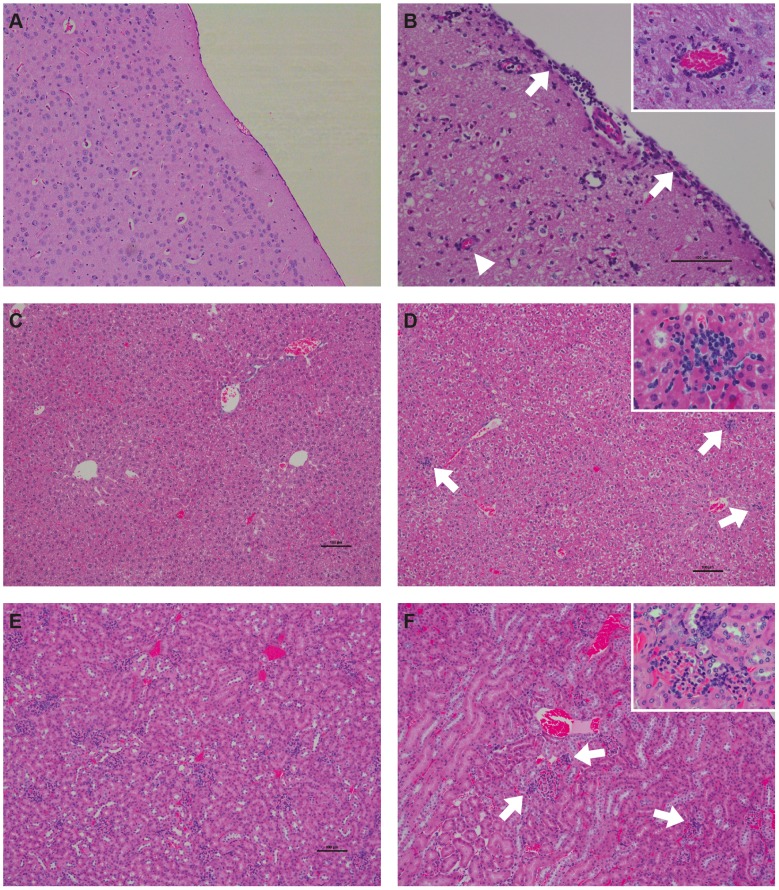
Histopathologic changes in BALB/c mice experimentally infected with KFDV P9605 and AHFV Zaki-1. (A and B) H&E stain of the brain at day 8 post-infection. (A) Mock. (B) KFDV P9605-infected mouse. Meninges are expanded by an inflammatory infiltrate (arrows), perivaculitis is characterized by infiltrations of mononuclear cells and neutrophils (inset). (C and D) H&E stain of the liver at day 4 post-infection. (C) Mock. (D) AHFV Zaki-1-infected mouse. Scattered foci of inflammatory cells (arrows), foci are composed of macrophages and lymphocytes (inset). (E and F) H&E stain of the kidney at day 6 post-infection. (E) Mock. (F) AHFV Zaki-1-infected mouse. Congested kidney tissue and an increased cellularity of glomeruli (arrow head) with mononuclear inflammatory cells in proximity (inset).

**Figure 5:**
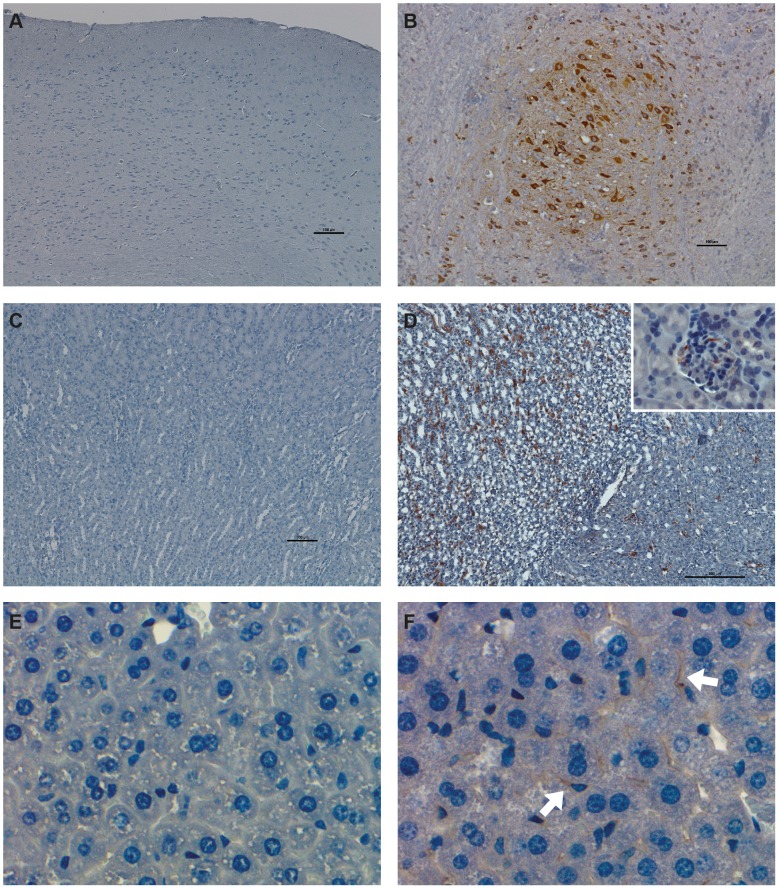
Immunohistochemical findings in BALB/c mice experimentally infected with KFDV P9605 and AHFV Zaki-1. (A and B) Immunohistochemistry of the brain. (A) Mock. (B) Foci of positive immunostaining for KFDV P9605 (brown) within neurons and some astrocytes at day 8 post-infection. (C and D) Immunohistochemistry of the kidney at day 4 post-infection. (C) Mock. (D) AHFV Zaki-1-infected mouse, viral antigen (brown) can be found scattered in the cortex and the medulla. Positive immunostaining for AHFV Zaki-1 in mesangial cells of the glomeruli (inset). (E and F) Immunohistochemistry of the liver on day 6 post-infection. (E) Mock. (F) AHFV Zaki-1-infected mouse. AHFV Zaki-1 immunostaining (brown) in sinusoids and Kupffer cells (inset).

In the brain, lesions were first observable at day 6 in some of the animals and were characterized by mild meningitis with infiltrates primarily composed of lymphocytes (data not shown). KFDV P9605 immunoreactivity was observed in the brains of two of the five animals euthanized at this time (data not shown). Positive immunostaining was observed in scattered neurons of the superficial cerebral cortex. At day 8 brain lesions were more widespread and severe (Figure 4B). Meninges were expanded by inflammatory infiltrates including lymphocytes, plasma cells, and neutrophils (Figure 4B). There were few migrating inflammatory cells directly underlying the affected pia mater within the molecular layer of the cerebral cortex. There was occasional perivascular cuffing and endothelial hypertrophy. KFDV P9605 immunoreactivity was observed in the brains of all animals at day 8 within scattered foci of neurons and occasionally in astrocytes (Figure 5B). Interestingly, meninges and affected vessels were commonly devoid of antigen. Two animals showed mildly increased cellularity of alveolar walls in the lung at day 8 due to infiltration of macrophages and lymphocytes in localized foci (data not shown). Antigen could be detected sporadically by immunohistochemistry in these foci. No lesions or antigen were detected in the liver, spleen, or kidney of mice infected with KFDV P9605.

### Histopathology of AHFV Zaki-1 and immunohistochemical detection in the liver, kidney, and spleen

Small, scattered foci of inflammatory cells were detected throughout the liver of most of the animals infected with AHFV Zaki-1 at days 6 and 8 post-infection. Foci were composed of macrophages and lymphocytes (Figure 4D). Interestingly, viral antigen could not be detected in these foci but was rather found in Kupffer cells lining the sinusoids (Figure 5F). Kidneys appeared congested and an increased cellularity of the glomeruli was noted at day 4 in all animals (Figure 4F). AHFV Zaki-1 immunoreactivity was observed in the kidneys of three out of the five animals from days 2 through 6 post-infection (data not shown). AHFV Zaki-1 immunostaining was observed in cells of the distal cortex, outer and inner medulla (Figure 5D), and sporadically in the mesangial cells of the glomeruli (Figure 5D). At day 4 there was dropout of lymphocytes in the germinal centers of the spleen and increased numbers of macrophages. By day 6 there was pronounced reactive hyperplasia. Immunoreactivity for AHFV Zaki-1 was observed in scattered mononuclear cells in three animals at day 2, one at day 4, and two at day 6 post-infection (data not shown). Antigen was detected within mononuclear cells scattered throughout the parenchyma. No lesions or antigen was detected in the brains of mice infected with AHFV Zaki-1. There were also no lesions or antigen detected in any of the tissues harvested from mock-infected control mice.

### AHFV Zaki-1 induces hematologic changes despite causing no disease

**Figure 6:**
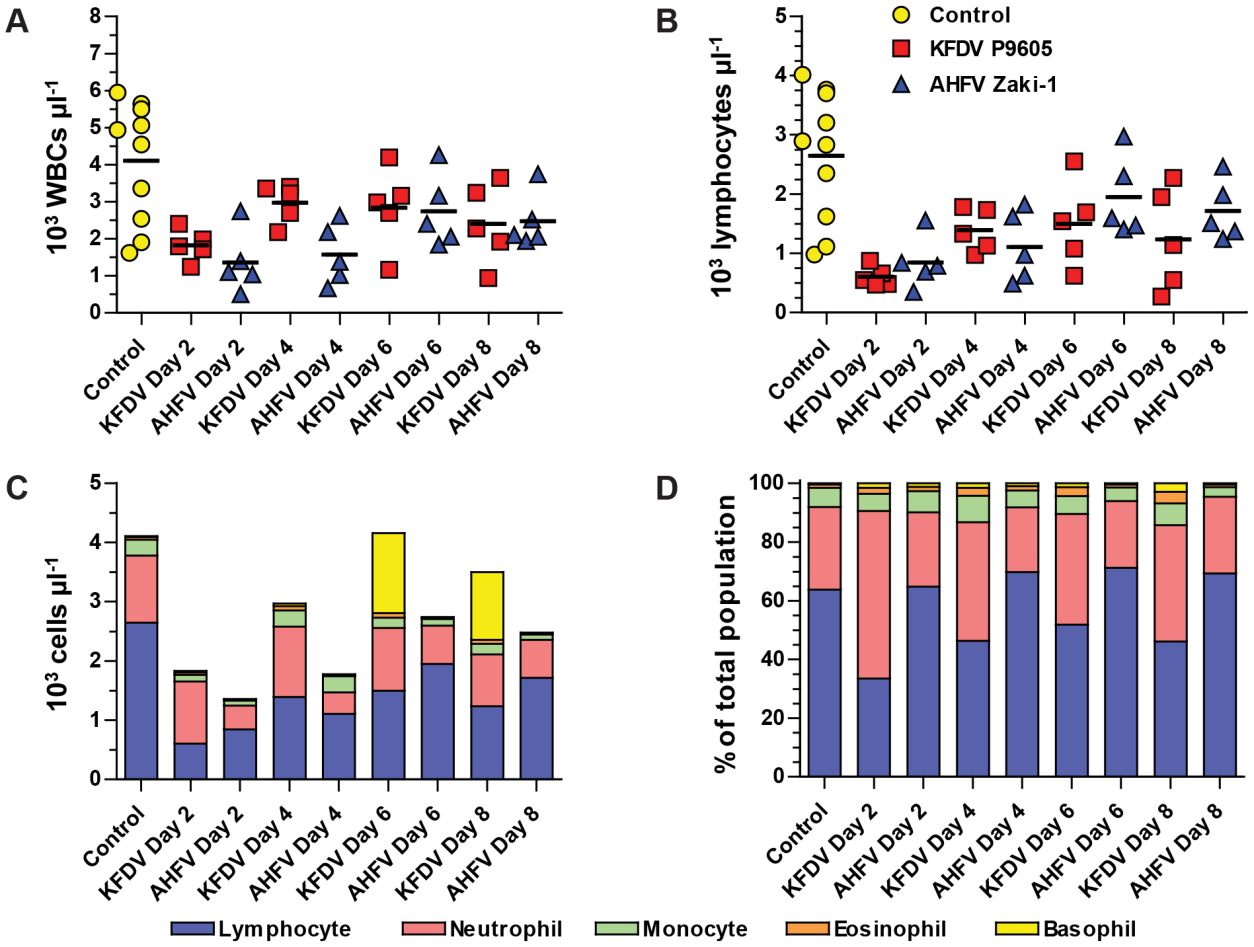
White blood cell (WBC) abnormalities induced by KFDV P9605 and AHFV Zaki-1 infection. (A) Total WBC counts from whole blood. Each symbol represents one animal. (B) Lymphocyte counts from whole blood. Each symbol represents one animal. Arithmetic means in (A) and (B) are indicated by horizontal black lines. (C) Composite graph showing average absolute counts of lymphocytes, neutrophils, monocytes, eosinophils, and basophils from 5 mice per day for each virus, and 10 uninfected control animals. (D) Composite graph showing average population proportions for lymphocytes, neutrophils, monocytes, eosinophils, and basophils from 5 mice per day for each virus, and 10 uninfected control animals.

**Figure 7:**
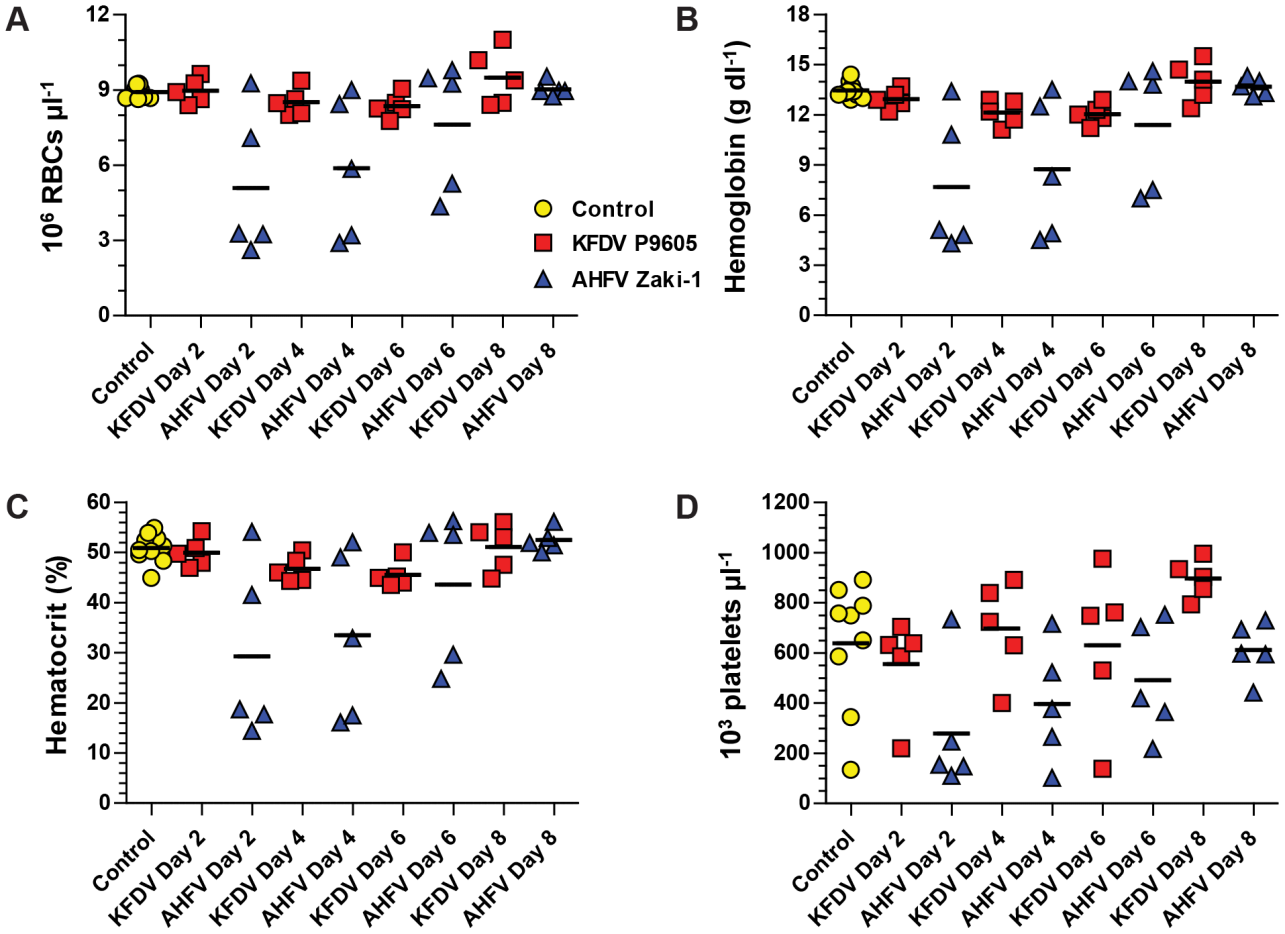
Hematologic abnormalities induced by KFDV P9605 and AHFV Zaki-1 infection. (A) Total RBC counts from whole blood. (B) Hemoglobin (Hb) levels from whole blood. (C) Hematocrit levels from whole blood. (D) Platelet counts from whole blood. Each symbol represents one animal, and arithmetic means are indicated by horizontal black lines.

We further characterized the responses of mice to KFDV P9605 and AHFV Zaki-1 by evaluating panels of hematological and clinical chemistry parameters. Both KFDV P9605 and AHFV Zaki-1 induced leukopenia on day 2 post-infection, although these levels recovered partially in mice infected with KFDV P9605 after this point (Figures 6A and 6C). The recovery of white blood cell levels was delayed until day 6 in mice infected with AHFV Zaki-1 (Figures 6A and 6C). There was a specific decrease in lymphocyte levels in mice infected with both viruses and these levels recovered over the course of the study (Figures 6B and 6C). The proportion of white blood cells (lymphocytes, monocytes, neutrophils, basophils, and eosinophils) in mice infected with AHFV Zaki-1 remained stable, whereas lymphocyte proportions decreased and neutrophil proportions increased in mice infected with KFDV P9605 (Figure 6D). AHFV Zaki-1-infected mice also had decreased red blood cells counts (Figure 7A), hemoglobin (Hb) levels (Figure 7B), hematocrit (Figure 7C), and platelet counts (Figure 7D), whereas these parameters remained stable in KFDV P9605-infected mice. Thus, despite a lack of clinical disease, AHFV Zaki-1 induces several of the hallmark signs of hemorrhagic fever observed in other animal models [36]–[39].

### AHFV Zaki-1 and KFDV P9605 induce pro-inflammatory cytokines in target organs

**Figure 8:**
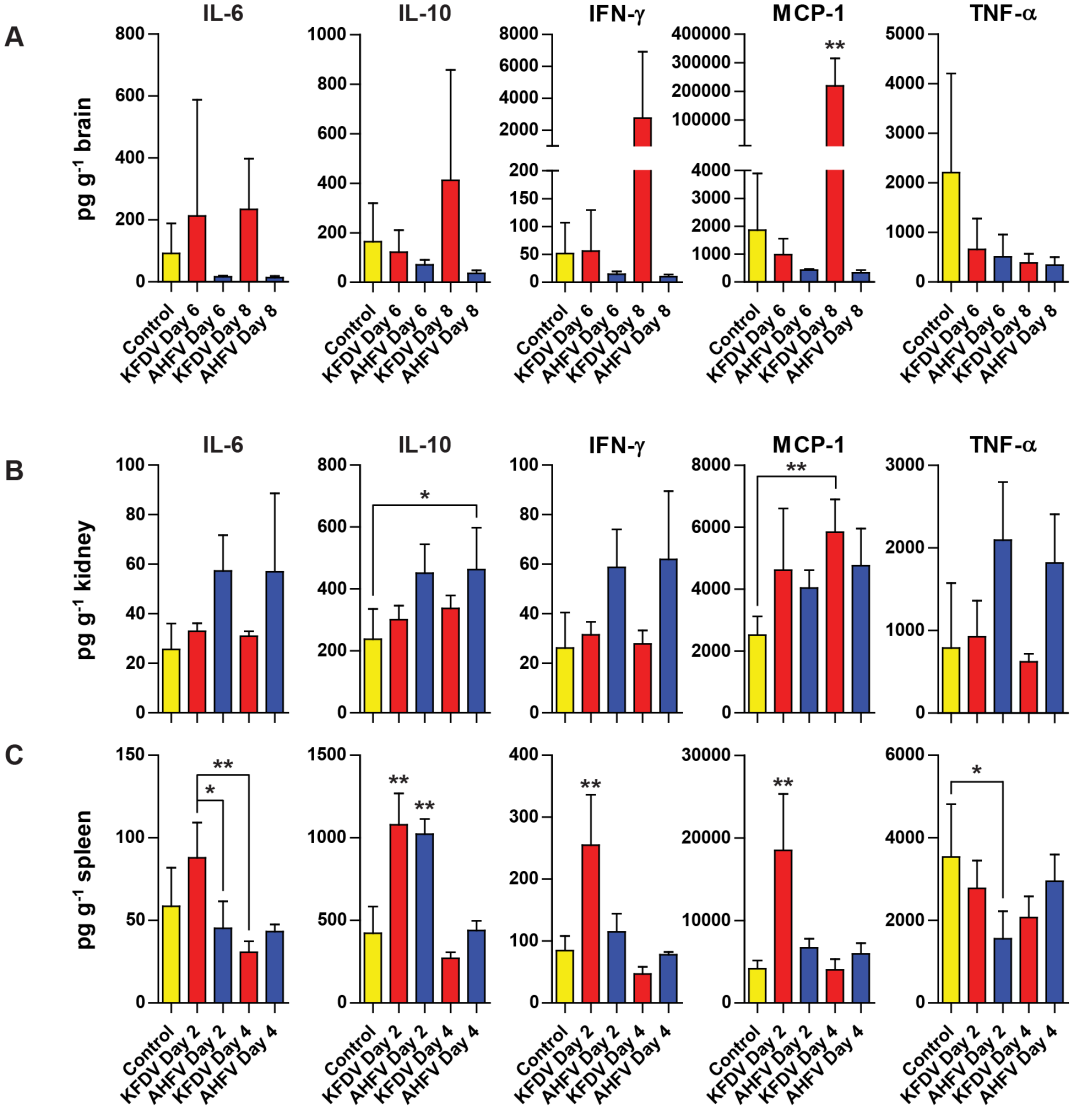
Differential cytokine induction by KFDV P9605 and AHFV Zaki-1. Organ homogenates were analyzed by Bio-Plex Pro Mouse Cytokine Assay as described in the Materials and Methods. Results for IL-6, IL-10, IFN-γ, MCP-1, and TNF-α are shown for the brain (A), kidney (B), and spleen (C). All cytokine concentrations were normalized to the amount of the respective tissue that was homogenized in each sample. Error bars represent standard deviations. Statistical significance is indicated by * for *p*<0.05 and ** for *p*<0.01.

We have already shown that the highest KFDV P9605 virus titers were present in the brain at days 6 and 8 post-infection, while no AHFV Zaki-1 was detected in the same tissue (Figure 3B). Likewise, AHFV Zaki-1 virus titers are the highest at day 2 and 4 in the kidney and spleen, but KFDV P9605 was not detected in this organ (Figures 3C and 3D). We therefore analyzed the homogenates of these organs from the indicated time points by Bio-Plex Pro Mouse Cytokine Assay for a panel of cytokines. In the brain, KFDV P9605 induced large increases in the amount of IL-10 and IFN-γ, and MCP-1 at day 8, while IL-6 levels were elevated at both days 6 and 8 (Figure 8A). Mice infected with AHFV Zaki-1 had levels of IL-6, IL-10, IFN-γ, and MCP-1 in the brain that were lower than those found in the control or KFDV P9605 animals (Figure 8A). The levels of TNF-α in the brain were generally lower than what was found in the control animals for both viruses (Figure 8A). AHFV Zaki-1 infection induced the production of IL-6, IL-10, IFN-γ, MCP-1, and TNF-α in the kidney at days 2 and 4 post-infection, while the response to KFDV P9605 infection was similar to control animals for these cytokines, with the exception of significantly elevated levels of MCP-1 on day 4 (Figure 8B). Finally, infection with KFDV P9605 also resulted in a significant induction of IL-6, IFN-γ, and MCP-1 in the spleen on day 2, while both KFDV P9605 and AHFV Zaki-1 induced significantly higher levels of IL-10 (Figure 8C). However, the levels of these cytokines diminished rapidly by day 4 for both viruses (Figure 8C). The levels of TNF-α were lower than in control animals on day 2 after AHFV Zaki-1 infection but increased by day 4 (Figure 8C), while the levels of IL-6 and IFN-γ were unchanged on both days (Figure 8C). Both KFDV P9605 and AHFV Zaki-1 are therefore able to induce pro-inflammatory cytokines in organs where virus replication is detected, although the inflammatory response in the spleen is brief.

## Discussion

To the best our knowledge, we have performed the first study that systematically examines the pathogenesis of the tick-borne flaviviruses KFDV and AHFV in an immunocompetent mouse model. KFDV P9605 was lethal in BALB/c mice, but did not cause disease consistent with a hemorrhagic fever. The AHFV strains 2003 and Zaki-1, in contrast, were not lethal in mice and did not cause any overt clinical disease, but the pattern of virus detected in organs (kidney, liver, and spleen), and the observed hematologic abnormalities (leukopenia, lymphopenia, and decreased hematocrit) were consistent with some aspects of hemorrhagic fever.

### Is KFDV neurological infection and lethality a result of adaptation?

Previous studies of tick-borne flavivirus pathogenesis have used either of the immunocompetent mouse strains BALB/c or C57BL/6 mice [26]–[28] and the Bogoluvovska strain of OHFV, which has approximately two suckling mouse brain (SMB) passages, and extensive tissue culture passages [26]. This virus caused enlarged spleen and some evidence of hemorrhages in the liver, as well as rapid onset of clinical signs with death occurring around day 9 post-infection [26]. Of particular note is the lack of neurological disease and detection of virus only within the cerebellum of infected animals [26]. Further studies with OHFV strain Guriev resulted in high virus titers in the brain and relatively low titers in other peripheral organs [27]. Although KFDV P9605 does not have as many SMB passages as OHFV Guriev (9 compared to 24 for OHFV Guriev), we found that its distribution in tissues was similar to the results of Tigabu *et al.* [27], namely that the highest virus titers were found in the brain, but only at low levels elsewhere. This suggests that this neuroinvasive phenotype is perhaps due to neuroadaptation over the course of many SMB passages. In prior studies, mice infected with OHFV Guriev showed some signs of mild neurological disease [27], but we did not observe comparable signs in mice infected with KFDV P9605. KFDV with higher 9 SMB passages (strain 1639, which is similar to KFDV P9605 used in our experiments) has also been found in the CSF of infected macaques at later stages of disease [29], supporting the conclusion that KFDV may have acquired a neuroinvasive phenotype during SMB passage. The practice of passaging virus isolates in mouse brains was common in the 1950s before the widespread availability of tissue culture, so it is consequently quite challenging to obtain virus isolates that have not been extensively passaged in mice.

### AHFV infection induces hematologic abnormalities and pro-inflammatory cytokines with no overt signs of clinical disease

We found virus distributed in tissues commonly associated with hemorrhagic fever (kidneys, liver, and spleen) in mice infected with AHFV Zaki-1, which appeared at early time points, but was then cleared. We did not find significant amounts of virus in the brain or lungs, which also corresponds with what has been observed for OHFV [26]. Similar to what has been observed by others upon OHFV infection, AHFV induces a pro-inflammatory cytokine response early in the kidney and spleen, and differences in some hematologic parameters, namely leukopenia combined with decreases in lymphocytes, RBC count, hemoglobin levels, and hematocrit [28], indicating that AHFV Zaki-1 causes a sub-clinical hemorrhagic-like syndrome. Lethal infection of mice with TBEV is not associated with elevated body temperatures [40], while animals infected with KFDV in our study had transient fever during the later phase of disease. This suggests that tick-borne flaviviruses that are primarily associated with neurological disease do not induce fever. The presence of KFDV in the brain may therefore reflect a spillover event into the CNS due to factors such as increased vascular permeability rather than a true neuroinvasive phenotype as is seen with TBEV infection. Initial studies with AHFV showed that it was lethal in suckling mice when injected intracerebrally (IC) or intraperitoneally (IP), and in adult mice when administered via the IP route [8]. This discrepancy likely reflects the fact that injection by the IC and IP is a more efficient means of infecting mice compared to footpad injection, which we used for our experiments. We infected mice IP with AHFV Zaki-1, but the dose was not sufficient to cause disease or death. Footpad injection also requires a much smaller volume of inoculum than other routes, so higher doses of virus are not always practical or even possible.

### Lack of plasma viremia in tick-borne flaviviruses

We were unable to detect virus in the plasma of infected animals for both KFDV P9605 and AHFV Zaki-1. This confirms the findings of previous studies, which also failed to detect free virus in animals infected with other tick-borne flaviviruses [27], [28]. It is unclear why there is no detectable viremia in infected mice, especially since we found that there was generally more virus in the *in vitro* culture supernatant than in the cellular fraction (see Figure 1). We suspect that the virus is associated with circulating leukocytes (macrophages or dendritic cells), as has been described for other viruses such as morbilliviruses [41], [42] and henipaviruses [43]. Interestingly, another study found that KFDV was lethal in bonnet macaques (*Macaca radiata*) and high virus titers were detected in the serum [29], which suggests that the lack of free virus in the serum may be restricted to rodent species.

In this study we evaluated the pathogenesis of KFDV and AHFV in the BALB/c mouse model. Here we found that AHFV Zaki-1 infection did not cause a clinically evident disease but showed viral tropism, hematological changes, and pro-inflammatory cytokine response suggestive of a viral hemorrhagic fever. In contrast, infection with KFDV was uniformly lethal with evidence of a neuro-inflammatory disease. Histopathology and immunohistochemistry findings support the tissue distribution phenotype of KFDV P9605 and AHFV Zaki-1 observed by titration of virus in organs, and demonstrates the invasiveness of these viruses in the respective tissues. KFDV P9605 seems to invade the brain on or before 6 days post-infection and causes moderate meningitis, with virus replicating in neurons in all areas of the brain. Surprisingly, KFDV P9605 replication could not be detected in any other visceral organs, with the exception of the lung. AHFV Zaki-1 was found in a range of visceral tissues where it only causes mild histopathologic changes. It should be noted that although there is no detectable viremia, AHFV Zaki-1 immunoreactivity was found in multiple tissues with filtering function such as glomeruli in the kidney, Kupffer cells in the liver, and macrophages in the spleen. Despite finding AHFV Zaki-1 antigen and infectious virus in multiple organs, mice appear to be able to control and eliminate the virus effectively. These data also suggest that KFDV is more neurotropic than AHFV and support the hypothesis that KFDV infection can cause neurological disease despite its infrequent occurrence in humans. It is possible that serial passage of KFDV P9605 in the brains of suckling mice contributed to the enhanced neurovirulence. Clearly, the use of authentic low-passage KFDV isolates would be desirable for this study to empirically evaluate the neurovirulence of KFDV. Unfortunately, low-passage KFDV strains were not available to us. Future studies will examine the influence of progressive virus passage of AHFV and KFDV in different organs on disease presentation.
